# Fluorosis varied treatment options

**DOI:** 10.4103/0972-0707.62631

**Published:** 2010

**Authors:** I Anand Sherwood

**Affiliations:** Reader, Department of Conservative Dentistry, CSI College of Dental Sciences, Madurai, India

**Keywords:** Dental fluorosis, endemic fluorosis areas, fluorosis, treatment options for fluorosis

## Abstract

Fluorosis has been reported way back in 1901. The treatment options for fluorosis are varied depending upon individual cases. This article comes from Madurai in India where its surrounding towns are fluorosis-prone zones. The purpose of this article is to report various treatment options available for dental fluorosis; this is the first time that complete full mouth rehabilitation for dental fluorosis is being reported. This article also dwells on the need for the dentists to be aware of their local indigenous pathologies to treat it in a better manner.

## INTRODUCTION

It is well documented that fluoride can have both beneficial and detrimental effects on the dentition ever since Mc Kay and G.V. Black in 1916 published the effect of fluoride on dentition.[1] The beneficial effects of fluoride on dental caries are due primarily to the topical effect of fluoride after the teeth have erupted in the oral cavity. In contrast, detrimental effects are due to systemic absorption during tooth development resulting in dental fluorosis.[2] The purpose of this article is to report various treatment options available for dental fluorosis; this is the first time that complete full mouth rehabilitation for dental fluorosis is being reported.

Credit for the identification of the scientific basis for the fluoride in preventing and treating dental caries is largely attributed to the work of two American dentists - Dr. Fredrick Mckay and an US public health officer H. Trendley Dean.[1] It was Dean, 1934, who developed a classification for fluorosis, which is still widely used, based on his interpretation of clinical appearance.[3] Dean and Mckay suggested that optimum level of water fluoride should be below 0.9 - 1.0 PPM.[4]

In India, fluorosis was identified in 1937 in Nellore of Andhra Pradesh by Shortt *et al*.[5] Geological crust of India, especially South India, has fluoride rich bearing minerals which can contaminate underground aquifers.[6] Nearly 73% of Tamil Nadu is hard rock crust.[7] In Tamil Nadu, Madurai is a known endemic fluorosis area and has fluoride level in drinking water of about 1.5 - 5.0 ppm.[8]

The earliest manifestation of dental fluorosis is an increase in enamel porosity along the striae of Retzius.[9] Clinically, the porosity in the subsurface of enamel reflects as opacity of the enamel. With an increased exposure to fluoride during tooth formation, the enamel exhibits an increased porosity in the tooth surface along the entire tooth surface. Very severely hypo mineralized enamel will be very fragile and hence as soon as they erupt into oral cavity they undergo surface damage as a result of mastication, attrition and abrasion. The definite evidence that fluoride can induce dental fluorosis by affecting the enamel maturation was given by Richards *et al*.[10] Thylstrup and Fejerskov proposed a way of recording dental fluorosis (TF index) based on the histopathological features.[11] Human and animal studies have shown that the enamel hypomineralization in fluorotic teeth are due to aberrant effects of fluoride on the rates at which enamel matrix protein breakdown or rates at which the byproducts of enamel matrix degradation are withdrawn, resulting in retardation of crystal growth in enamel maturation stage.[12]

Dean's index:[3]

1 – Questionable - occasional white fleckings and spottings of enamel

2 – Mild - white opaque areas involving more of the tooth surface

3 – Moderate and severe - pitting and brownish staining of tooth surface

4 – Corroded appearance of tooth

TF score:[11]

0 – Normal translucency of the glossy creamy white enamel remains after wiping and drying of the surface

1 – Thin white lines are seen across the tooth surface

2 – Opaque white lines are more pronounced and frequently merge to form small cloudy areas scattered over the whole surface of the tooth

3 – Merging of white lines occurs, and cloudy areas of opacity occurs spread over many parts of the surface. In between the cloudy areas, white lines also can be seen

4 – The entire surface exhibits a marked opacity or appears chalky white

5 – The entire surface is opaque and there are round pits

6 – The small pits frequently merge in the opaque enamel and forms bands

7 – There is loss of outer surface of enamel in irregular areas and less than half the surface is involved

8 – The loss of outer most enamel surface is more than half the enamel

9 – The loss of major part of the outer enamel results in change of anatomical shape of the tooth

Other indices available are:

- The tooth surface fluorosis index[13]
- Fluorosis risk index[14]

Treatment options for fluorosis varies with severity.[15] Depending upon severity, treatment option varies:

- Micro/Macro abrasion
- Bleaching
- Composite restorations
- Veneers
- Full crowns

## CASE REPORTS

### Case A

**Figure 1:**
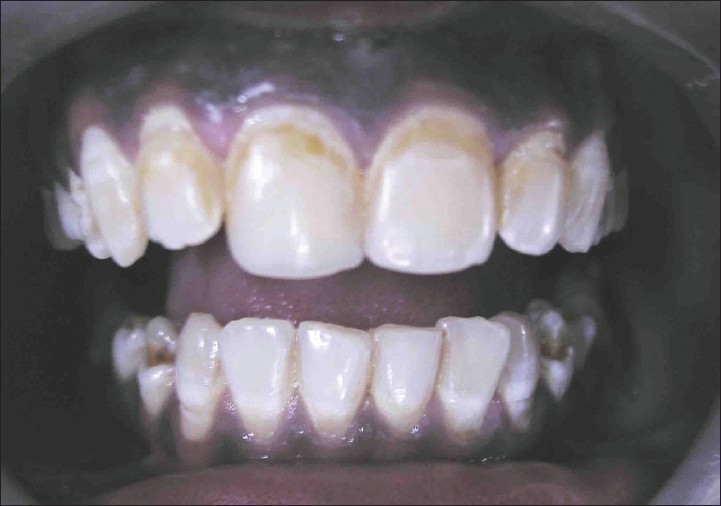
Preoperative (case A)

A patient named Sudhakar, aged 21, with a chief complaint of discolored upper front teeth [Figure 1] reported to department of conservative dentistry in our hospital. He gave a history of discoloration since his childhood. No other relevant medical history was elicited from him.

On examination he had mild grade of fluorosis according to Dean's Fluorosis index in his teeth 13 - 23 and 33 - 43 [Figure 1]. All other teeth were present, no dental caries was found. His oral hygiene was poor.

**Figure 2:**
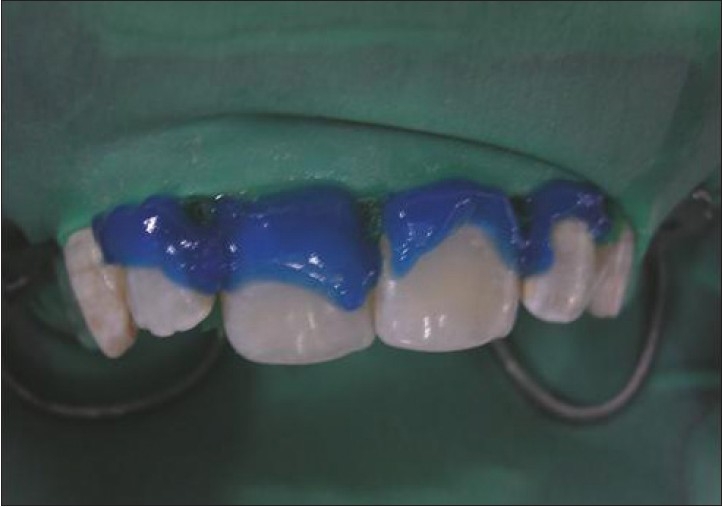
Etching followed by bleaching with McInnes solution (case A)

**Figure 3:**
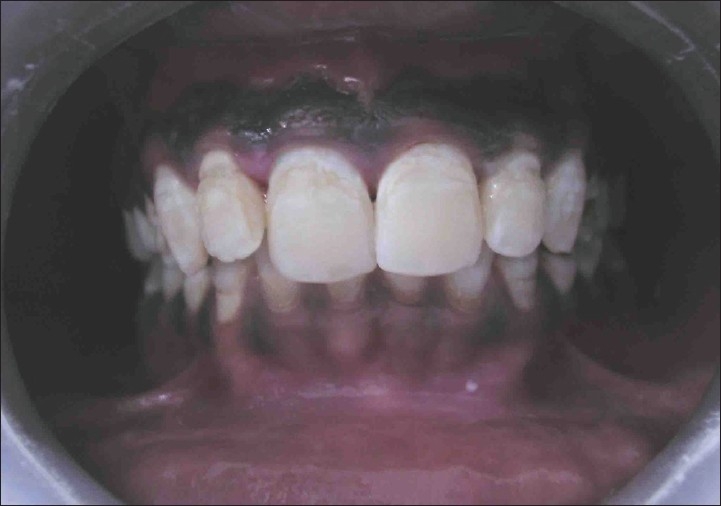
Post operative photo (case A)

The first phase of treatment involved oral prophylaxis. This was followed by the second phase of treatment. for in- office vital bleaching using McInnes solution[16] aided by etching of the teeth by 37% phosphoric acid.[17] Bleaching procedure was for three sittings [Figures 2 and 3].

### Treatment procedure summary

McInnes solution has been used for treating mild grade fluorosis for a long time[16] and successfully. In this patient, an in-office bleaching approach was advocated because of mild grade of fluorosis. McInnes solution consists of one part anesthetic ether, five parts hydrochloric acid (36%), five parts hydrogen peroxide (30%). The solution was freshly mixed and applied onto tooth using a cotton applicator. Each bleaching session consisted of application of bleaching solution for five minutes with one minute interval under rubber dam application followed by polishing of teeth with prophylaxis paste[18] viz., Proxit (Ivoclar Vivadent). The patient was satisfied with the outcome after three sittings, which were done a week apart from each session.

### Case B

**Figure 4:**
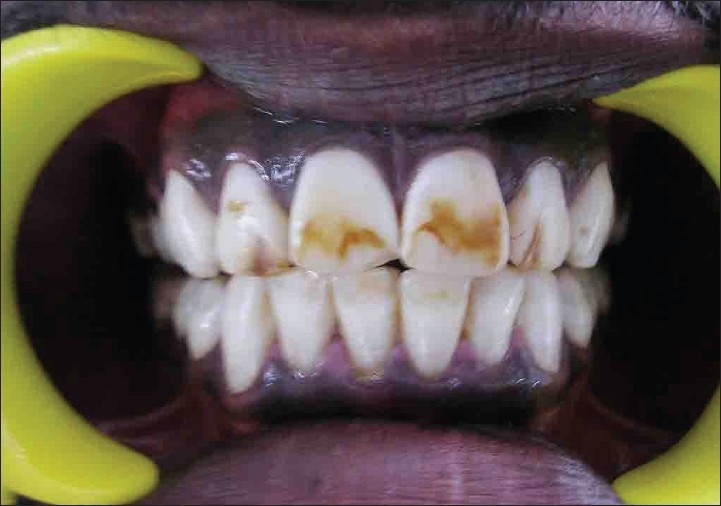
Preoperative (case B)

A patient named Deenadayalan, aged 25, with a chief compliant of discolored upper front teeth [Figure 4] reported to department of conservative dentistry in our hospital. Patient gave a history of discoloration since his childhood. No other relevant medical history was reported by the patient.

**Figure 5:**
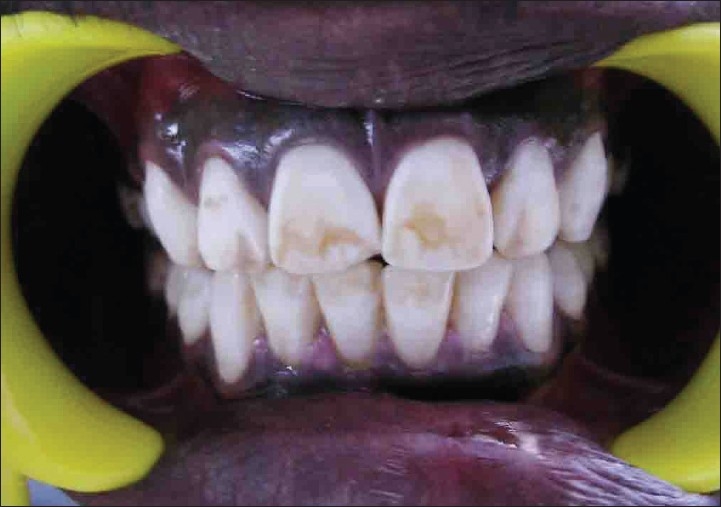
Post operative followed by macro and micro abrasion (case B)

On examination, mild grade fluorosis according to Dean's Fluorosis index was present in his teeth 13 - 23 and 33 - 43 [Figure 4]. Dental caries were present in his tooth 46 and 36. His oral hygiene was good. His dental caries were restored with amalgam restorations. Treatment plan involved micro and macro abrasion followed by polishing[17] [Figure 5].

### Treatment procedure summary

Micro and macro abrasion were employed for this patient. This technique has been employed successfully for mild to moderate grade fluorosis.[19] The tooth 12 - 22 were abraded using water cooled fine diamond finishing flame shaped points, with diamond abrasive particle size of 20 - 30*μ*m with a high speed hand piece to remove surface enamel layer of 0.5mm thickness. Removal of surface enamel was done with intermittent pressure under water coolant. Final polishing of teeth was done with polishing discs (Super Snap, Shofu Inc.,). The patient was satisfied with final aesthetic outcome.

### Case C

**Figure 6:**
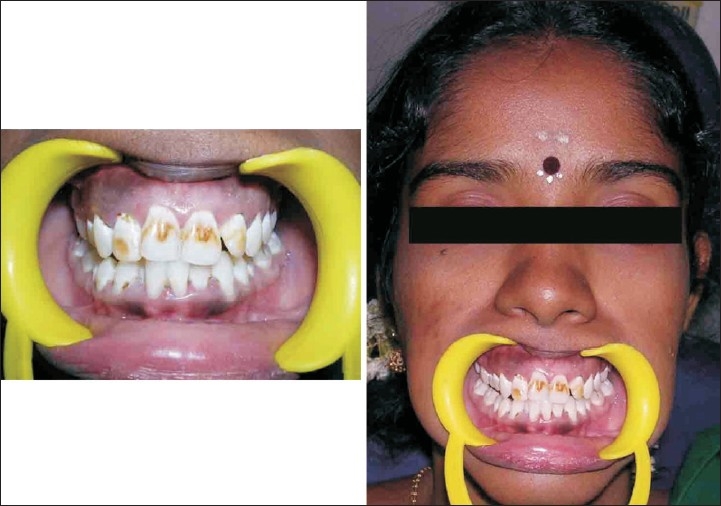
Preoperative (case C)

A patient named Sudha, aged 23, with a compliant of discolored upper front teeth [Figure 6] reported to department of conservative dentistry in our hospital. Patient gave history of discoloration present from her childhood. There was no relevant history reported by the patient.

On examination she had mild to moderate fluorosis in teeth 13 - 23 and 43 - 33 [Figure 6]. Her oral hygiene was poor. No dental caries was found in any of her teeth.

**Figure 7:**
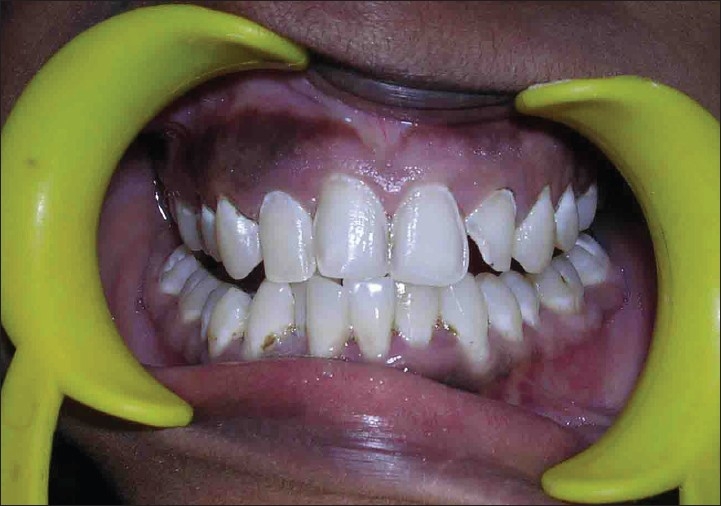
Tooth preparation for veneer restoration (case C)

**Figure 8:**
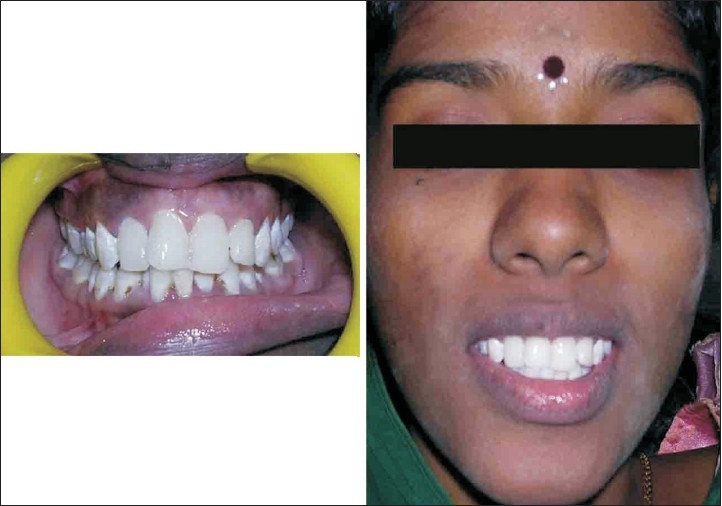
Post operative (case C)

Treatment plan involved direct composite restorations from 12 - 22, because of the time constraint give by the patient [Figures 7 and 8] and also presence of moderate grade of fluorosis, according to Dean's Fluorosis index.

### Treatment procedure summary

Composite resin has been used for treatment of dental fluorosis.[15] The treatment involved veneer preparation with window design,[17] composite resin used was nano-composite Ceram-X Duo (Dentsply, India) enamel shade E1 and dentin shade D2 bonding agent employed was Prime and Bond NT (Dentsply, India). Polishing of composite restoration was accomplished with Super Snap (Shofu Inc, Japan). The patient was satisfied with treatment outcome.

### Case D

**Figure 9:**
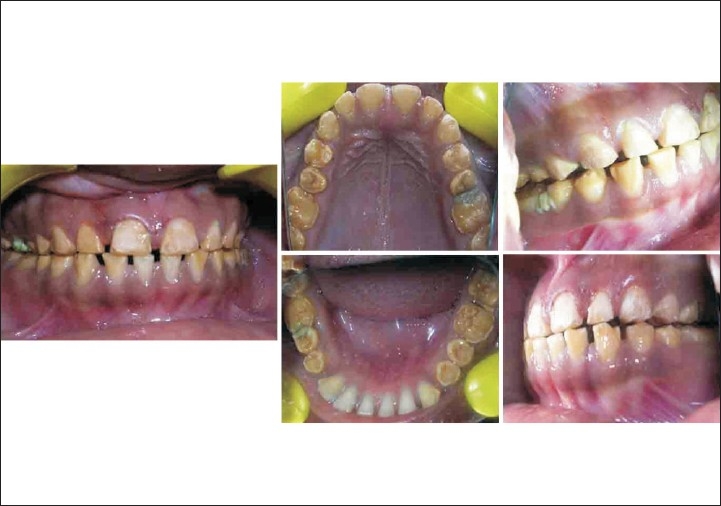
Preoperative (case D)

Patient named Illakya, aged 17, reported with a chief complaint of discolored teeth [Figure 9] reported to department of conservative dentistry in our hospital. Patient gave history of discoloration from her childhood. No relevant medical history was reported by patient.

On examination she was had moderate to severe fluorosis on all her teeth. Enamel in all of her teeth had been chipped off and all her molar teeth had undergone severe attrition with no cuspal inclines or morphology present. Class II glass ionomer restoration was found in teeth 26 and 45, 46. She also had loss of vertical dimension due tooth structure loss [Figure 9] as estimated by her inter-cuspal distance at rest being 4mm. Besides these intra-oral findings she gave negative findings for any temporo-mandibular joint dysfunction. Her oral hygiene was good.

Treatment plan involved full mouth rehabilitation with porcelain fused to metal crowns on all her teeth.

### Treatment procedure summary:

**Figure 10:**
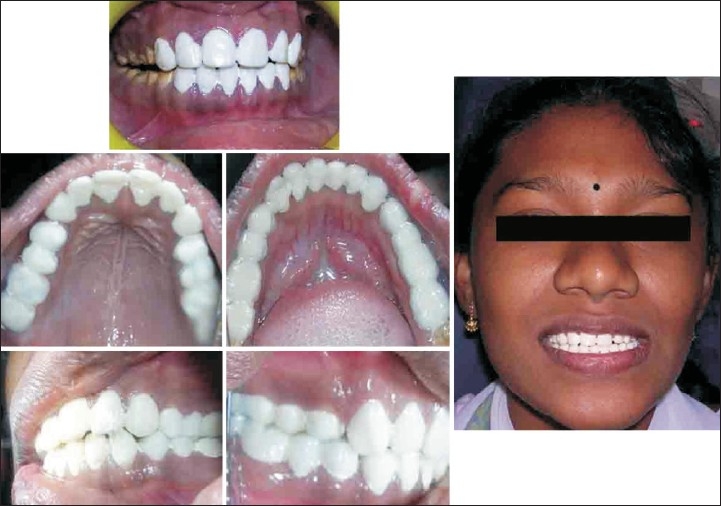
Temporarization (case D)

Pre operative impressions, occlusal records, face bow transfer were taken followed by occlusal wax up in pre operative mounted models. Anterior crown preparations were done followed by temporary crown insertion with increased vertical dimension and a new centric was recorded [Figure 10]. Anterior temporary crowns acted as a deprogramming device. Temporary crowns were fabricated with heat cure acrylic resin.[20–23]

Posterior crown preparations were done followed by temporary crown insertion in newly raised occlusal vertical dimension [Figure 10].

**Figure 11:**
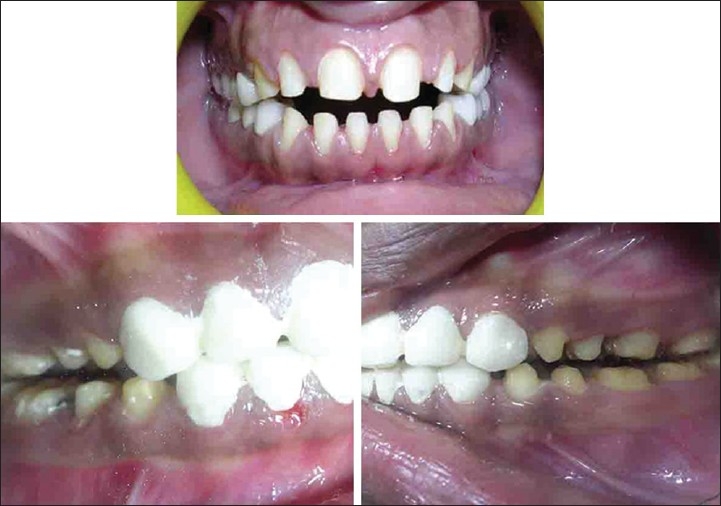
Tooth preparation and removal of temporary crowns (case D)

**Figure 12:**
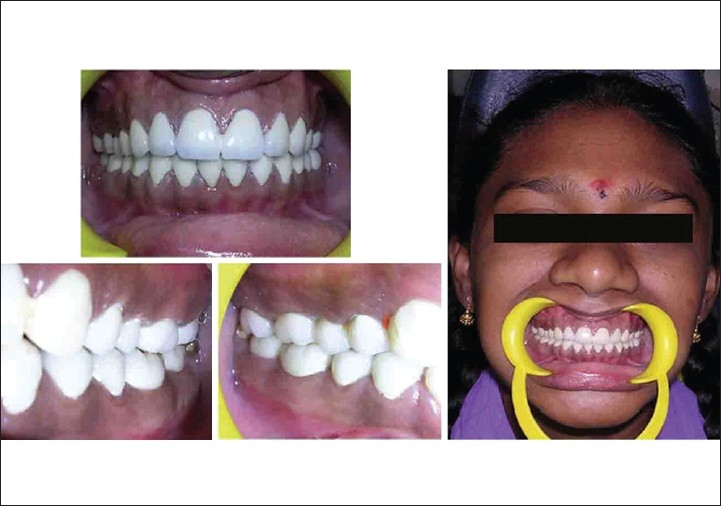
Postoperative (case D)

**Figure 13:**
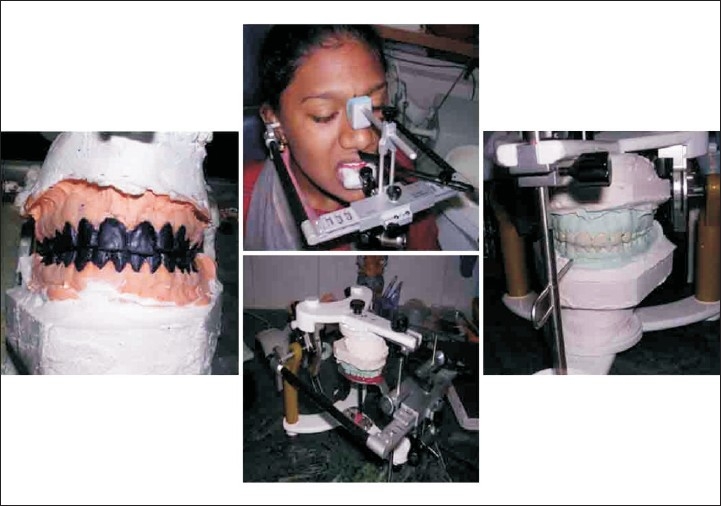
Wax up, face bow transfer and occlusal records (case D)

Posterior metal ceramic crowns were luted followed in next appointment by luting of the anterior metal ceramic crowns [Figures 11 and 12]. Articulators used in this case were Stratos 300 (Ivoclar - Vivadent) an Arcon type semi adjustable articulator and Universal face bow (Ivoclar - Vivadent) [Figure 13].

## DISCUSSION

In case A, patient had mild grade of fluorosis and therefore in-office vital bleaching procedure with McInnes solution was advocated. McInnes solution has been successfully used for treating mild fluorosis. Advantage of this procedure is that it is relatively non-invasive compared to other restorative procedures and also it could be done with minimum chair side time. The main disadvantage of this procedure is the postoperative sensitivity it produces and that it cannot be employed in patients with more severe grade of fluorosis.[24] Vital bleaching is more successful for fluorosis in younger patients presenting with opaque to orange colour stain rather than older patients with darker type of brown stains.[25]

In case B patient had mild grade fluorosis and micro and macro abrasion were employed. Advantage of micro and macro abrasion being its much faster procedure in achieving the desired result compared to other treatment options. However, the main disadvantage is that these procedures employ high speed rotary instrument which can lead to excessive removal of tooth structure is operator does not have the desired skill level.[17]

Abrasion techniques can be successfully employed for discoloration presented either as single line discoloration or patchy type of discoloration, it cannot be successfully employed for discoloration which is more diffuse in nature.[26] Both the bleaching technique and abrasion procedures could be employed only for mild to moderate grade fluorosis.[25,26] Most of the times, a combined treatment regimen of bleaching and abrasion procedures is employed to produce the desired aesthetic result in patients with yellowish discoloration due to fluorosis.[27]

In case C, the patient had moderate type of fluorosis which necessitated that the patient was treated by veneer procedure. Veneers have been successfully employed for management moderate grade fluorosis,[15] Because of the time constraint given by patient, direct composite veneer treatment option was selected. Advantage of direct composite veneer is that it is done with minimal chair time when compared to indirect ceramic veneers, disadvantage being its long term wear resistance, color stability.[17]

In case D, patient had loss of vertical dimension of occlusion and patient's inter-occlusal space was 4mm at rest. Therefore a treatment plan was evolved to increase the vertical dimension of occlusion by 4mm which was within the limit for the patient.[20] Full mouth restorations were planned with metal ceramic crowns. This treatment option of restoring vertical dimensions of occlusion for severe fluorosis patients requires careful investigations and preparation. This treatment option is limited to cases with severe fluorosis and loss of inter-occlusal space. Advantage of this procedure is that it is an extensive procedure by which the desired aesthetic results and functional efficiency is achieved. The main disadvantage is also its extensiveness in treatment procedure which requires extensive lab procedure and operator skill, knowledge.

In each of the treatment options described above, each one has its own advantages and disadvantages; a good clinician should be aware of all the treatment options available assess its merits and demerits and select the best treatment option according to individual patient needs.

## CONCLUSION

Fluorosis is a major health problem in India with over 65 million people at risk and 6 million children seriously affected.[28] Skeletal fluorosis, which is caused due to long term exposure of fluoride, does not seem to be a problem in Tamil Nadu with less than 1% prevalence.[29] Data clearly indicate the prevalence of fluorosis as an endemic problem for Tamil Nadu.

In all the cases described here, diagnosis of dental fluorosis was made from their familial history and place of residence and type of drinking water used. All the patients in this report were from localities in and around Madurai. No other contributory findings were elicited for discoloration of tooth due to other reasons. One of the most important parts of diagnosis of dental fluorosis is differentiating this entity from amelogenesis imperfecta and molar-Incisal hypo mineralization (MIH) and most important data for differentiating dental fluorosis from other pathologies will be familial history, place of residence, chronology of discoloration appearance.[30] In spite of all these findings, dental fluorosis is difficult to distinguish clinically and histologically from other type of hypoplastic and hypomineralized enamel.[31]

The purpose of this article was to report various treatment options available for dental fluorosis from a conservative bleaching management to extensive full crown restorations and this has been the first time that complete full mouth rehabilitation for dental fluorosis been reported. Also, it has been reported from findings that the predominant cariostatic effect of fluoride is not due to its uptake by the enamel during tooth development but during cyclic de- and re-mineralization processes, which take place at the tooth/oral fluid interface so it's possible to achieve caries reduction without concomitant risk of dental fluorosis.[12] So it is in the interest of both patient and dentist that the dentist be aware of all the treatment modalities available to us. Newer treatment options which combine these various treatment modalities are emerging.[32,33] Other treatment options available are laser assisted bleaching, abrasion employing abrasive pastes.[32,33] This article does not advocate that one treatment option is superior to another but rather the severity of the lesion alone determines the treatment option.
